# Nanostructured Thin Coatings Containing *Anthriscus sylvestris* Extract with Dual Bioactivity

**DOI:** 10.3390/molecules25173866

**Published:** 2020-08-25

**Authors:** Irina Negut, Valentina Grumezescu, Alexandru Mihai Grumezescu, Alexandra Cătălina Bîrcă, Alina Maria Holban, Iuliana Urzica, Sorin Marius Avramescu, Bianca Gălățeanu, Ariana Hudiță

**Affiliations:** 1 Lasers Department, National Institute for Lasers, Plasma and Radiation Physics, 077125 Magurele, Romania; negut.irina@inflpr.ro (I.N.); valentina.grumezescu@inflpr.ro (V.G.); iuliana.iordache@inflpr.ro (I.U.); 2 Department of Science and Engineering of Oxide Materials and Nanomaterials, Faculty of Applied Chemistry and Materials Science, Politehnica University of Bucharest, 011061 Bucharest, Romania; ada_birca@yahoo.com; 3 Microbiology & Immunology Department, Faculty of Biology, University of Bucharest, 077206 Bucharest, Romania; alina_m_h@yahoo.com; 4 University of Agronomic Science and Veterinary Medicine, 59 Marasti Blvd., 011464 Bucharest, Romania; sorin_avramescu@yahoo.com; 5 Department of Organic Chemistry, Faculty of Chemistry, Biochemistry and Catalysis, University of Bucharest, 90–92 Soseaua Panduri, 050159 Bucharest, Romania; 6 Department of Biochemistry and Molecular Biology, Faculty of Biology, University of Bucharest, 91–95 Splaiul Independentei, 050095 Bucharest, Romania; bianca.galateanu@bio.unibuc.ro (B.G.); arianahudita@yahoo.com (A.H.)

**Keywords:** thin films, *Anthriscus sylvestris*, magnetic nanoparticles, antimicrobial, multifunctional

## Abstract

Plant extracts are highly valuable pharmaceutical complexes recognized for their biological properties, including antibacterial, antifungal, antiviral, antioxidant, anticancer, and anti-inflammatory properties. However, their use is limited by their low water solubility and physicochemical stability. In order to overcome these limitations, we aimed to develop nanostructured carriers as delivery systems for plant extracts; in particular, we selected the extract of *Anthriscus sylvestris* (AN) on the basis of its antimicrobial effect and antitumor activity. In this study, AN-extract-functionalized magnetite (Fe_3_O_4_@AN) nanoparticles (NPs) were prepared by the co-precipitation method. The purpose of this study was to synthesize and investigate the physicochemical and biological features of composite coatings based on Fe_3_O_4_@AN NPs obtained by matrix-assisted pulsed laser evaporation technique. In this respect, laser fluence and drop-casting studies on coatings were performed. The physical and chemical properties of laser-synthesized coatings were investigated by scanning electron microscopy, while Fourier transform infrared spectroscopy comparative analysis was used for determining the chemical structure and functional integrity. Relevant data regarding the presence of magnetic nanoparticles as the only crystalline phase and the size of nanoparticles were obtained by transmission electron microscopy. The in vitro toxicity assessment of the Fe_3_O_4_@AN showed significant cytotoxic activity against human adenocarcinoma HT-29 cells after prolonged exposure. Antimicrobial results demonstrated that Fe_3_O_4_@AN coatings inhibit microbial colonization and biofilm formation in clinically relevant bacteria species and yeasts. Such coatings are useful, natural, and multifunctional solutions for the development of tailored medical devices and surfaces.

## 1. Introduction

Cancer is one of main causes of death worldwide, and the number of cancer-affected patients is ever-increasing [1,2]. Colorectal cancer is the third most common type of cancer in the US and the second leading cause of cancer deaths; it was estimated that new cases of colorectal cancer will reach ~133,000/year in the next five years [3,4]. The direct involvement of microorganisms in cancer has been known for over a century [5]. Bacteria are promoters of oncogenic processes, as they produce toxins that interfere with cellular signals, therefore disturbing the regulation of cell growth [6]. Colorectal cancer has been in the center of epidemiological research, with the main scope being the determination of causative factors for this disease. Several scientific studies have focused on the part played by microbes in colorectal cancer etiology [7,8]. For example, evidence has shown that colonic adenomas, carcinomas, and the mucosa of colorectal cancer are inhabited by high numbers of adhering *Escherichia coli* of phylotype B2 [9,10,11]. It has been implied that the presence of some *E. coli* strains in colonic cancer development is associated with chronic inflammation—a consequence of bacterial infection that effects both the host and microbiota, specifically by stimulating the spread of *E. coli* [12]. Moreover, bacteremia is a life-threatening complication in patients with cancer, especially those that are subjected to chemotherapy, because of the secondary effects of this type of treatment: ulcerative lesions in mucosal surfaces and weakened host defenses. For example, epidemiological data suggest that intestinal colonization by the opportunistic pathogen *Pseudomonas aeruginosa* is prominent among hospitalized and cancer patients; the intestinal carriage of *P. aeruginosa* rises from ~3% in healthy individuals to up to ~30% in hospitalized patients [13]. On the other hand, several studies have analyzed the influence of fungal diseases on cancer establishment and progression. *Candida albicans* has been the most studied due to the fact that it can disseminate hematogenously in immunosuppressed cancer patients and spread to multiple organs [14].

Consequently, progressive methods aiming to offer both a constant and targeted release of antibiotics together with a cancer treatment are crucial.

Recently, the advent of infection-producing pathogens with resistance to antibiotics and their association into biofilms has pressed the medical community to research and discover new substances with antimicrobial activity. The most investigated biofilm-embedded microorganisms are Gram-positive bacteria (such as *Staphylococcus aureus*), Gram-negative bacteria (*P. aeruginosa*, *E. coli*, etc.), and yeasts (like *C. albicans*) [15].

The increasing cost of current treatments (surgery, chemotherapy, and radiation) and the shortage of operative drugs has encouraged individuals to consider medicinal plant use [16]. Even though synthetic chemistry is now the leading method to synthesize drugs, the potential of plants and/or their extracts to offer novel products and means for disease prevention and treatment is still vast [17,18]. Plant use and traditional remedies in the management of various illnesses are as old as civilization [19] but still play an active role in typical treatments [20]. It has been estimated that ~50% of prescription products in Europe and the USA originate from natural products and their derivatives [21,22].

Plant products are available and affordable sources of biologically active agents that may have dissimilar mechanisms of action as compared with conventional drugs [23,24,25,26] and can represent foundations for the development of new medications for diseases like cancer [27,28,29,30] and microbial infection [31,32,33,34,35]. Therefore, new natural drugs can be advanced from plant essential oils and extracts as no microorganism resistance or adaption to these natural drugs has been demonstrated in recent years [36]. Phytochemicals isolated from medicinal plants have been screened for antibacterial and antitumor properties. These classes of natural drugs include polyphenols, taxol analogues, vinca alkaloids, and podophyllotoxin analogues [37].

Polyphenols have been shown to possess beneficial biological activities, such as antioxidant, anti-inflammatory, antiviral, and anticarcinogenic activities. Although polyphenols, organic chemicals which contain phenol units in their structures, have been shown to play important roles in many treatments, they are still limited as pharmaceutical products; a key problem is their low bioavailability under *in vivo* conditions [38]. Besides poor permeability, numerous polyphenols are metabolized in the gut and liver [39], consequently restraining the curative effect of their administration. Therefore, the formulation of phytochemicals in drug delivery systems capable of enhancing the absorption of active compounds is a necessary step.

*Anthriscus sylvestris* (Apiaceae family) (AN), commonly identified as wild chervil or cow’s parsley, is a wild biannual plant, mostly found in temperate regions [40]. Traditionally, AN has been used as an antipyretic, analgesic, diuretic, and as a cough remedy [41]. The chemical composition of AN has been assessed in numerous phytochemical studies on all plant organs (leaves, flowers, and roots). The major classes of phytochemicals include monoterpenes, phenylpropanoids, coumarins, and flavonoid lignans [42]. Previous studies have reported that various fractions of AN extract may have a potent antimicrobial effect against pathogens and opportunistic bacteria such as *Escherichia coli*, *Staphylococcus aureus*, and *Helicobacter pylori* [42]. *N*-hexane, methylene chloride (MC), ethyl acetate, and butanol fractions were proven to have antibacterial roles, while *n*-hexane and MC fractions inhibited the growth of *H. pylori* and gastric adenocarcinoma cells [42]. Furthermore, the antiproliferative effects of the roots and aerial part of AN showed a high in vitro inhibitory activity against MK-1, HeLa, and B16F10 tumor cell growth [43]. These results show that AN extract may have a multifunctional effect as a potent antimicrobial and anticarcinogenic agent.

Magnetic nanoparticles (NPs), in particular, magnetite (Fe_3_O_4_) nanoparticles, have attracted special attention in the biomedical field, especially in the design of antimicrobial and anticancer approaches. Fe_3_O_4_ nanoparticles are used as active transporters, tolerating drug transfer, and in targeted therapeutic effects [44,45,46]. In order to protect the magnetic core of this type of nanoparticle against corrosion, prevent phagocytosis by macrophages, and avoid the removal by reticuloendothelial systems, these nanoparticles need to be coated. The coating of magnetic nanoparticles with biodegradable and biocompatible polymers may overcome the poor control in drug adsorption and release associated with Fe_3_O_4_ nanoparticles [47,48]. Besides, sole magnetic nanoparticles are limited in carrying drug loads because of they lack the ability to control the amount of the drug and the rate of drug release. Conversely, biodegradable polymers have the ability to release loaded drugs at a rate dependent on their degradation [49]. Poly(lactide-co-glycolic acid) (PLGA) is a biocompatible and biodegradable polymer, and its application for human use in drug delivery systems of various therapeutic agents has been approved by the US FDA (Food and Drug Administration) and EMA (European Medicines Agency) [50,51]. In recent years, PLGA–NP systems have been some of the most studied due to their capacity for encapsulating drugs and biomolecules for applications in drug delivery [52,53].

Laser-based approaches for assembling thin films are used for the synthesis of these active bio-platforms aimed at delivering natural drugs for various applications, such as cancer and antimicrobial therapy. Matrix-assisted pulsed laser evaporation (MAPLE) is an additive laser deposition technique developed for functionalizing solid substrates with different types of coatings [54]. MAPLE provides high experimental versatility in terms of the possibility to preserve material properties (even for very delicate compounds) and by providing uniform and adherent coatings on different substrates [55]. This method allows the preparation of various drug concentrations to be immobilized as thin layers on the substrate material.

The purpose of this study was to develop and characterize a nanostructured thin coating based on PLGA, Fe_3_O_4_, and AN extract with multifunctional bioactivity. The main focus of this study was on assessing the antimicrobial activity of the coating against model microbial strains of opportunistic pathogens with clinical relevance (the Gram-positive *Staphylococcus aureus*, the Gram-negative *Escherichia coli*, and the yeast *Candida albicans*); the toxicity of this coating against tumor cells *in vitro* was also assessed.

## 2. Results and Discussion

### 2.1. Chromatographic Assay of Hydroalcoholic Extracts

**Figure 1:**
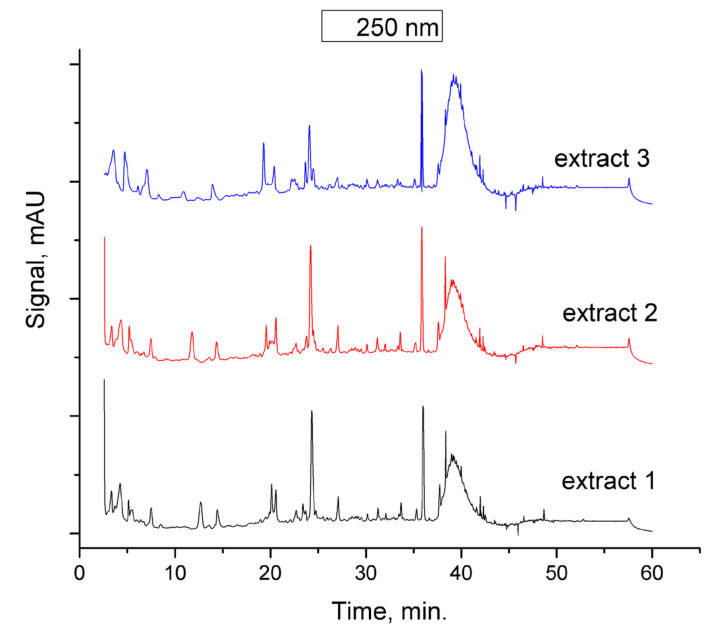
HPLC chromatograms of EtOH:H_2_O (70:30 *v*:*v*) *Anthriscus sylvestris* extract with detection at 250 nm.

Our findings by means of HPLC-DD method revealed the presence of nine polyphenols. The results of phenolic content are presented in Table 1, and the typical chromatogram can be observed in Figure 1. The analysis of AN and its main polyphenolic components, taking into account their medical efficiency, is essential. The identified polyphenols in the AN extract, especially those in high concentrations, present a diversified antitumoral activity in terms of decreasing tumor growth, cell cycle, and metastasis and increasing the drug response [56,57]. It was found that these compounds largely inhibited the growth of MCF-7, SK-BR-3, and ZR-75-1 cells in a dose-dependent manner [58]. Naringenin has been applied as an original treatment for cancer, as it suppresses cell growth and induces cell apoptosis of HT-29 colon cancer [59]. It was reported that epicatechin was effectively used to inhibit the growth of different cancer cells, such as those of prostate cancer, papilloma, and colorectal cancer [60]. Moreover, chlorogenic acid is a polyphenol that is able to change the behavior of tumor cells and induce cancer differentiation [61], representing an encouraging way to treat cancer.

The antioxidant activity of the studied extract was 99.6%. The incontestable antioxidant activity has a synergetic effect in conjunction with anticancer capacity. The chromatographic profile of the extracts spanning from polar (98% water) to less polar (100% ACN) indicates that most of the polyphenols are efficiently separated. Even if not all are identified, they surely contribute significantly to the targeted objectives. Reproducibility of the extraction procedure is ensured by the traceability of all involved steps: plant source (cultivated at Hofigal SA and collected in the same conditions), plant particle dimensions (<0.3 mm), certified solvents for mixture preparation, microwave extraction method, and HPLC analysis. The results (Figure 1 and Table 1) show that the chemical profiles of all three microwave-assisted extracts were similar.

A typical chromatogram of the obtained extract is presented in Figure 1.

### 2.2. Physicochemical Characterization of Fe_3_O_4_@AN Nanoparticles

**Figure 2:**
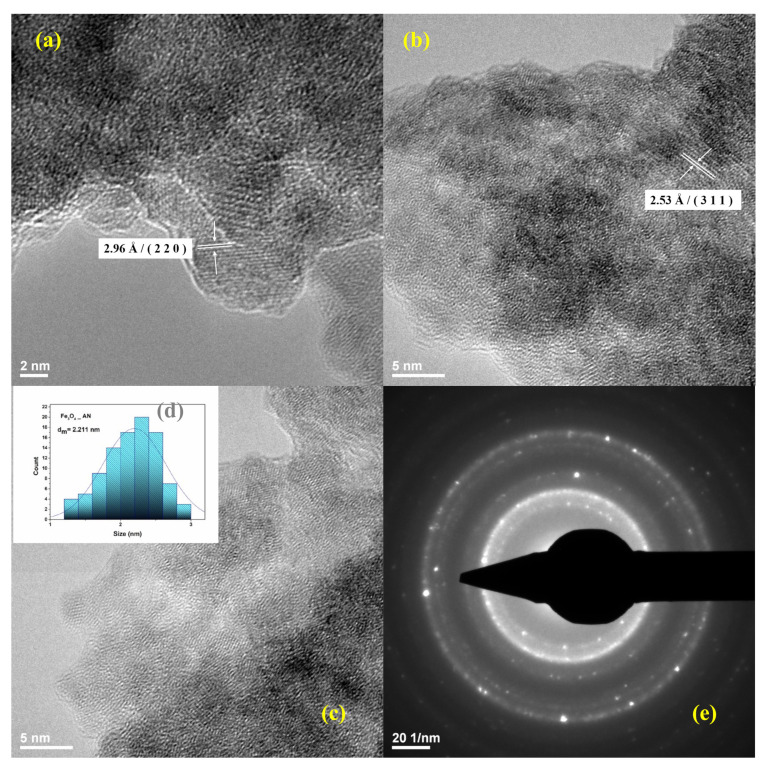
TEM images of Fe_3_O_4_@AN powder (**a**–**c**), histogram (**d**), and SAED pattern (**e**).

Fe_3_O_4_@AN nanopowders were characterized by TEM and SAED to show both the presence of Fe_3_O_4_ as the only crystalline phase and the size of NPs. In Figure 2a–c, a general tendency to aggregate is observable. Each aggregate is composed of a group of nanoparticles. The average size of NPs evaluated by TEM (Figure 2d) was measured to be ~2.2 nm. The SAED pattern (Figure 2e) confirms the crystalline structure of the Fe_3_O_4_@AN powder. Moreover, SAED patterns reveal a face-centered cubic structure of the investigated powder. The identified (2 2 0), (3 1 1), (4 0 0), (4 2 2), (5 1 1), and (4 4 0) diffraction planes are comparable with the data found in the literature [62,63] and correspond to ICDD (International Centre of Diffraction Data) card No. 19-0629.

### 2.3. Physicochemical Characterization of PLGA–Fe_3_O_4_@AN Coatings

**Figure 3:**
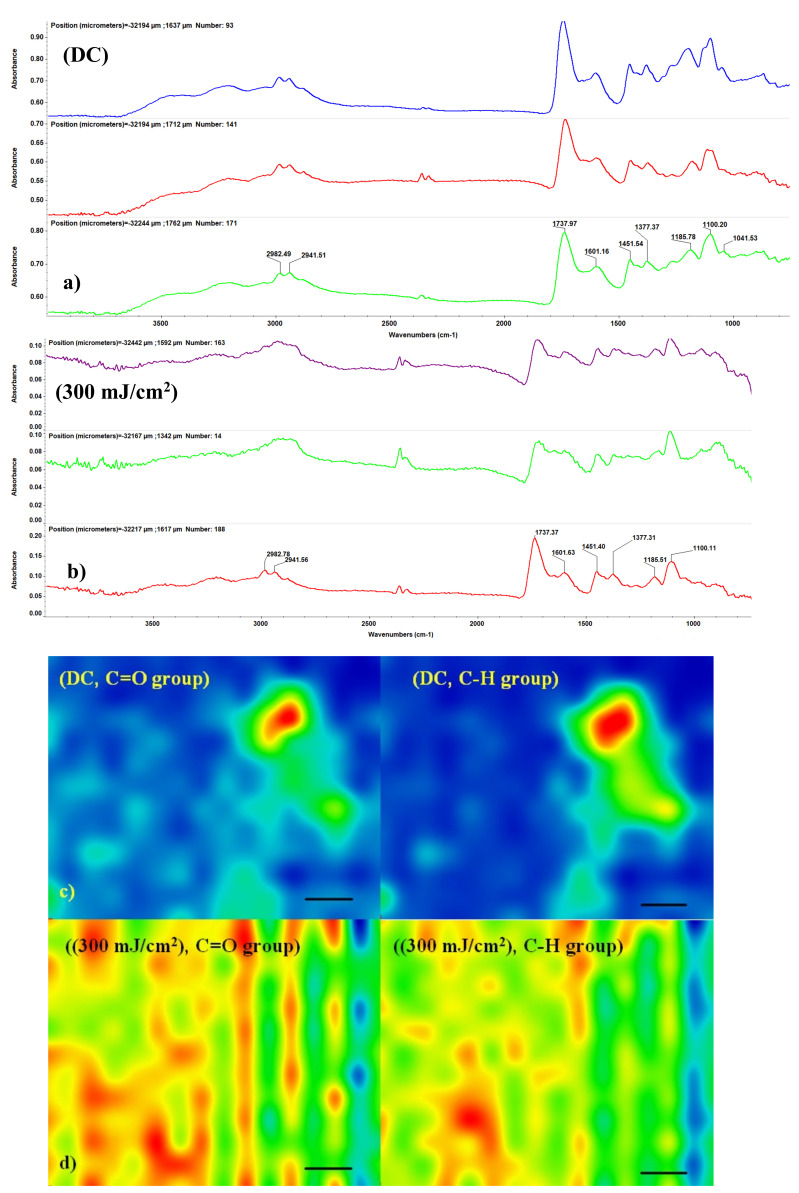
IR spectra of (**a**) drop-cast PLGA–Fe_3_O_4_@AN and (**b**) PLGA–Fe_3_O_4_@AN coatings obtained at 300 mJ/cm^2^; IR maps of (**c**) drop-cast samples and (**d**) thin coatings (scale bar 50 µm).

In order to examine the compositional integrity of laser-processed materials, we conducted comparative IR studies on the drop-cast (DC) samples PLGA–Fe_3_O_4_@AN vs. MAPLE coatings (Figure 3a,b). From the IR spectra of samples subjected to drop-casting (Figure 3a) and MAPLE deposition at 300 mJ/cm^2^ (Figure 3b), we can observe that a stoichiometric transfer was achieved. As a general remark, the IR spectra of MAPLE coatings synthesized at F = 300 mJ/cm^2^ resemble the corresponding drop-cast IR spectra.

Absorbance intensities of IR spectra maps are proportional to color alternations, beginning with the blue color (corresponding to the lowest intensity), gradually increasing through green and yellow, and finally reaching red (the highest intensity) [64]. The infrared spectrum of PLGA–Fe_3_O_4_@AN samples, both for coatings and drop-cast samples, is pictured in Figure 3. The weak infrared maxima identified at ~2941 and ~2980 cm^−1^ are attributed to the CH_3_ stretch. The peaks identified at ~1185 and ~1451 cm^−1^ can be attributed to C-O stretch groups and C-H stretch, respectively. The infrared peak identified at ~1737 cm^−1^ wavenumber is the consequence of C=O carbonyl group stretching [65,66]. The vibration peak identified at ~1041 cm^−1^ can be attributed to chain ester compounds of AN [67] and the C-O-C group from PLGA. As one can observe from the IR spectra maps, we obtained a structurally and compositionally homogenous coating. By associating this information with the classical IR analysis, it can be stated that the MAPLE deposition technique did not damage functional groups or induce changes to the chemical structure of the raw material.

**Figure 4:**
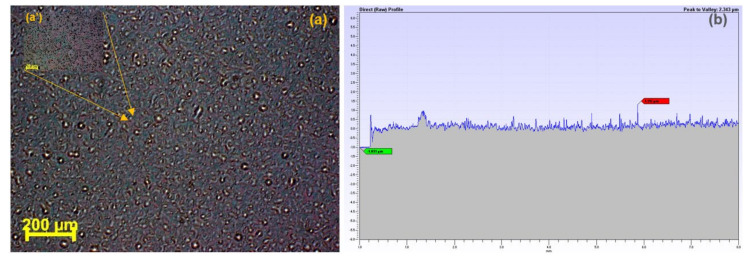
Optical microscopy images (in BF mode) at (**a**) 10× and (**a’**) 100× magnification; (**b**) thickness of the thin coating.

In order to determine the thickness of the MAPLE-deposited thin coating, the sample was scanned on an arbitrarily chosen distance of 8 mm. The thickness was 1175 ± 0.23 µm (Figure 4b). In the same image, one can observe the homogeneity of the thin coating. The surface roughness profile (XP2 Ambios surface profiler) was obtained by scanning the sample on a 5 mm surface; 60 individual scans were co-added and converted. The determined average roughness value, Ra, was 76 ± 0.16 nm.

### 2.4. Biological Evaluation of the PLGA–Fe_3_O_4_@AN Coatings

#### 2.4.1. In Vitro Toxicity of the PLGA–Fe_3_O_4_@AN Coatings against Human Adenocarcinoma HT-29 Cells

**Figure 5:**
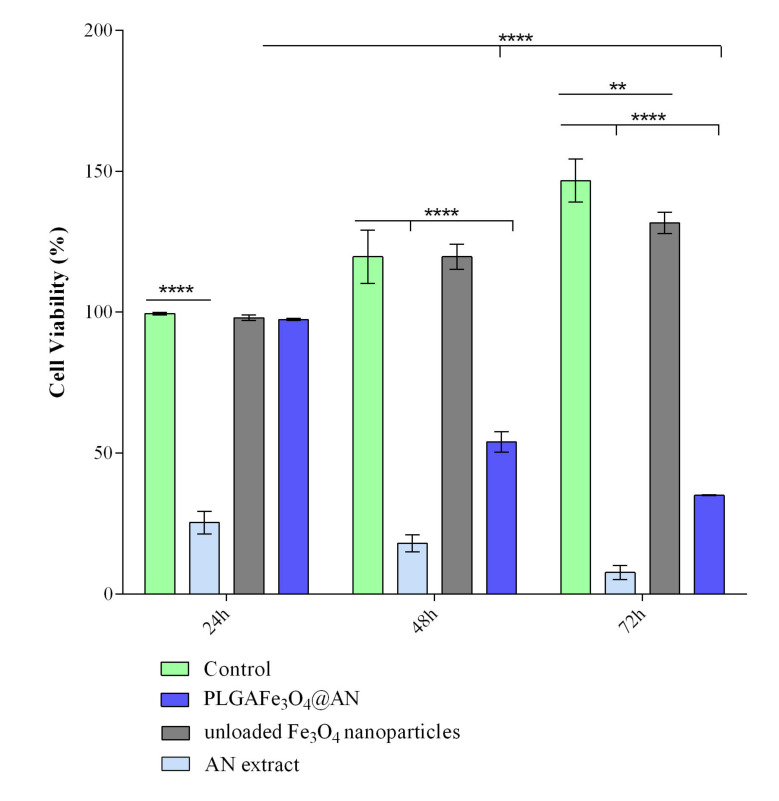
Cell viability of human adenocarcinoma HT-29 cells after 24, 48, and 72 h of contact with the PLGA–Fe_3_O_4_@AN coatings, as revealed by the MTT assay (** *p* ≤ 0.01, **** *p* ≤ 0.0001).

PLGA–Fe_3_O_4_@AN coatings were tested in order to investigate their ability to inhibit the cellular growth of the human adenocarcinoma HT-29 cells and to assess their in vitro cytotoxic potential overall. To accomplish this, the cellular metabolic activity of HT-29 cells in contact with the novel composites was evaluated by the MTT assay (Figure 5). After 24 h, no significant differences in cellular viability were observed in PLGA–Fe_3_O_4_@AN coatings as compared with the control glass slides. However, after 48 and 72 h, the cell viability of HT-29 cells was significantly (*p* ≤ 0.0001) decreased on the PLGA–Fe_3_O_4_@AN coatings as compared with the control samples. Moreover, a significant (*p* ≤ 0.0001) decrease of the cellular viability was observed for the PLGA–Fe_3_O_4_@AN coatings at 48 and 72 h as compared with 24 h, showing that the toxic effect of the coatings is considerably enhanced after prolonged exposure; therefore, the cellular viability decreases in a time-dependent manner. In contrast, the AN extract treatment triggered a dramatic decrease of the HT-29 cell viability, even after 24 h of treatment, where a 75% decrease of the cellular viability was observed (*p* ≤ 0.0001). After 72 h of treatment, only 7% of the treated HT-29 cells were viable, highlighting the high cytotoxicity of the AN extract to colon cancer cells when administered in free form. In order to assess if the simple magnetite carrier influences the cellular viability, HT-29 cells were exposed to pristine Fe_3_O_4_ treatment. In this case, a small decrease of the HT-29 cell viability was noticed only after 72 h of treatment, showing that the nanoparticles exhibit low cytotoxicity towards HT-29 cells.

**Figure 6:**
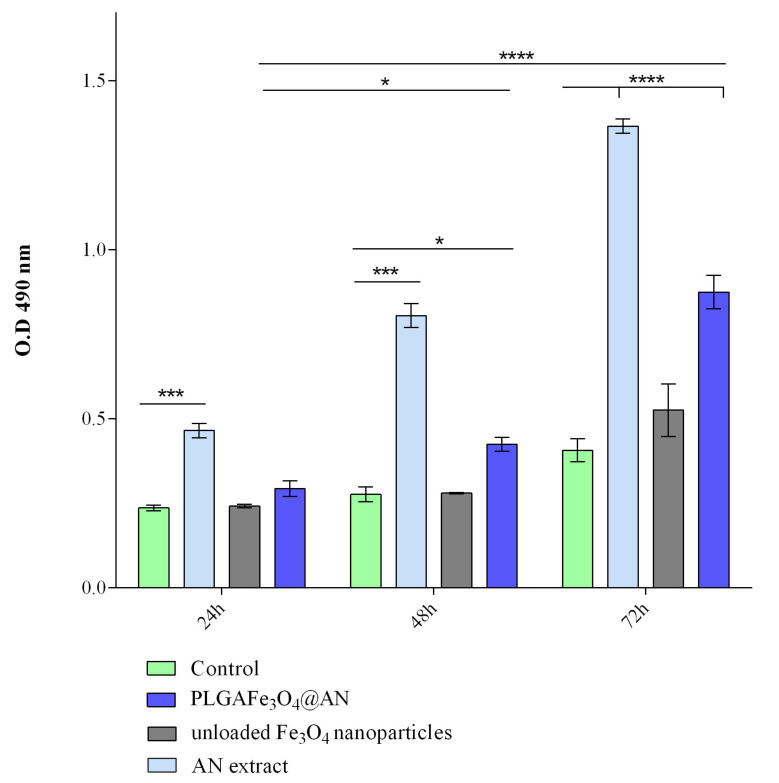
Cytotoxicity of the PLGA–Fe_3_O_4_@AN coatings, AN extract, and Fe_3_O_4_ nanoparticle treatment after 24, 48, and 72 h of culture, as revealed by the LDH assay (* *p* < 0.05; *** *p* ≤ 0.01; **** *p* ≤ 0.0001).

The cytotoxic potential of the PLGA–Fe_3_O_4_@AN coatings, AN extract, and Fe_3_O_4_ nanoparticles was investigated by measuring the LDH levels released in the culture media by damaged HT-29 tumor cells as a result of interaction with the composite’s surfaces or treatment exposure (Figure 6). After 24 and 48 h of culture, the PLGA–Fe_3_O_4_@AN coatings exhibited a low cytotoxicity, as the LDH activity was slightly increased as compared with the LDH levels detected in the control glass slides, with a statistically significant LDH level shift observed only after 48 h (*p* < 0.05). In contrast, 72 h postseeding, the LDH level released in the culture media was significantly (*p* ≤ 0.01) increased in PLGA–Fe_3_O_4_@AN coating samples as compared with control samples. Regarding the LDH activity in the culture medium of HT-29 cells treated with AN extract, our results were in full accordance with the MTT viability test. As presented, the AN extract treatment significantly increased the LDH activity, even after a 1-day exposure (*p* ≤ 0.001). In contrast, no statistically significant changes in the LDH activity were observed in HT-29 cell cultures treated with simple Fe_3_O_4_ nanoparticles, despite the slow increase of LDH levels after 72 h of treatment. Moreover, the LDH activity in the PLGA–Fe_3_O_4_@AN coating samples significantly increased over time as a rising profile of the LDH activity was detected at 48 and 72 h in comparison with 24 h, showing that the synthesized coatings present a long-term toxic effect on HT-29 tumor cells. Thus, our results highlight the cytotoxic potential of the novel synthesized PLGA–Fe_3_O_4_@AN coatings and also reveal that the HT-29 tumor cells are sensitive to the natural extract embedded in the original coatings, as prolonged contact with these severely affected the cell viability and metabolic activity of the HT-29 tumor cells.

#### 2.4.2. Microbiological Evaluation of the Coatings

The biofilm formation assay showed that infectious species presented dissimilar growth and capability to develop biofilms on laser-obtained coatings. Likewise, some variances in the biofilm-inhibitory action were detected at different time intervals.

The PLGA–Fe_3_O_4_@AN coatings seem to limit the formation and maturation of biofilms; various phases of biofilm development were altered, starting with cell attachment and initiation of biofilms and continuing with the maturation stages at 48 and 72 h, among the three tested species.

**Figure 7:**
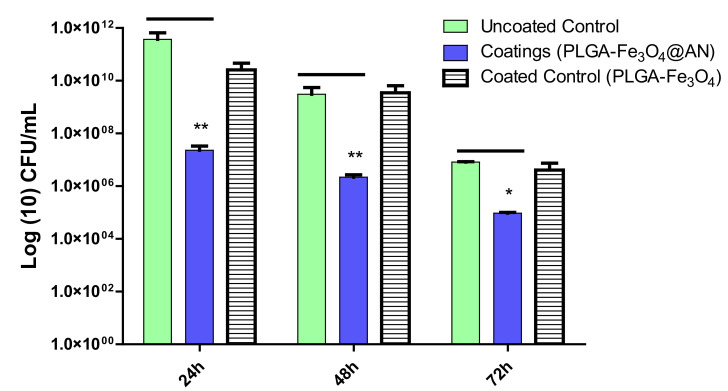
*S. aureus* biofilm formation (expressed as CFU (colony-forming units)/mL values) on control and coated surfaces for 24, 48, and 72 h of incubation at 37 °C (* *p* ≤ 0.05, ** *p* ≤ 0.01; comparison of coating versus control sample for the same time interval).

As visible from microbiological data, the most intense activity of PLGA–Fe_3_O_4_@AN coatings was demonstrated against the development of the *S. aureus* monospecific biofilms. When compared to the control (plain glass), the utmost effectiveness of PLGA–Fe_3_O_4_@AN coatings against early stages of *S. aureus* colonization and biofilm formation was detected after 24 h of incubation, since CFU/mL values decreased by more than 3 orders of magnitude. Furthermore, the efficiency of laser-obtained PLGA–Fe_3_O_4_@AN coatings against the *S. aureus* strain is sustained after incubation at 48 and 72 h (Figure 7).

**Figure 8:**
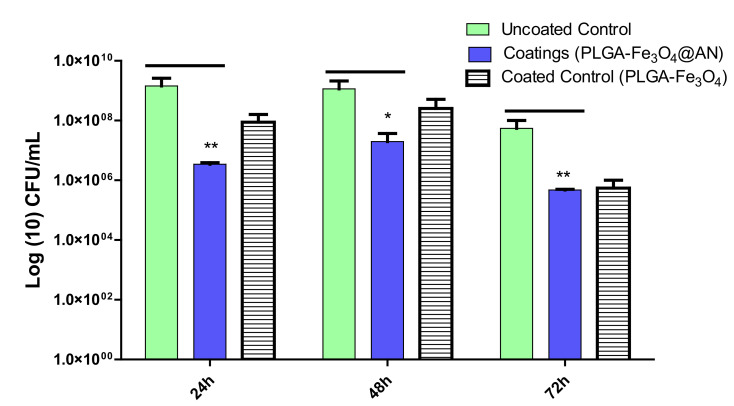
*E. coli* biofilm formation (expressed as CFU (colony-forming units)/mL values) on control and coated surfaces for 24, 48, and 72 h of incubation at 37 °C (* *p* ≤ 0.05, ** *p* ≤ 0.01; comparison of coating vs. control sample for the same time interval).

After 2 days of treatment, PLGA–Fe_3_O_4_@AN coatings demonstrated a slight inhibition behavior. The bacterial community was reduced by almost 1 order of magnitude. Although a slight relapse of bacterial biofilm formation is shown after 48 h, one can observe that CFU/mL values for biofilms at 72 h are reduced by more than 1 order of magnitude (Figure 8).

**Figure 9:**
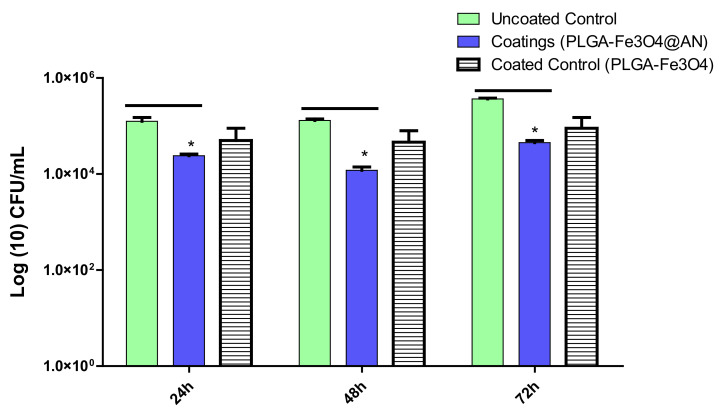
*C. albicans* biofilm formation (expressed as CFU (colony-forming units)/mL values) on control and coated surfaces for 24, 48, and 72 h of incubation at 37 °C (* *p* ≤ 0.05; comparison of coating versus control sample for the same time interval).

On the other hand, an attenuated antibiofilm activity was observed in the case of *C.* *albicans*, as CFU/mL values were reduced by a little over 1 order of magnitude for all time intervals (Figure 9).

Our obtained results support the antimicrobial efficiency of the AN extracts and show that the obtained coating can promote and ensure a prolonged activity of this extract for at least 72 h. Our results support previous findings regarding the efficiency of functionalized Fe_3_O_4_ NPs against biofilm formation [68]. The impact of polymers, such as PLGA, in stabilizing and increasing the efficiency of the bioactive NPs (probably by increasing the load of the bioactive compounds or extract) is also supported by this study [69,70].

## 3. Materials and Methods

### 3.1. Materials

The anhydrous ferric chloride (FeCl_3_ - anhydrous), poly(lactic-co-glycolic acid) (PLGA) with a 50:50 lactide:glycolide molar ratio, heptahydrate ferrous sulfate (FeSO_4_·7H_2_O), ammonia solution (NH_3_, 25%), methanol, and isopropanol were purchased from Sigma-Aldrich (Darmstadt, Germany). Substances necessary for biological assays, namely Dulbecco’s modified Eagle’s medium (DMEM), antibiotic antimycotic solution, 3-(4,5-dimethylthiazol-2-yl)-2,5-diphenyltetrazolium bromide (MTT), and TOX7 kit, were also purchased from Sigma-Aldrich (Darmstadt, Germany). Human colorectal adenocarcinoma cell line HT-29, *Escherichia coli* ATCC 25922 and *Staphylococcus aureus* ATCC 23235 bacterial strains, and *Candida albicans* ATCC 10231 fungal strain were acquired from the American Type Culture Collection (ATCC, Manassas, VA, USA).

The fetal bovine serum (FBS) was purchased from Life Technologies, Foster City, CA, USA. Tris (tris(hydroxymethyl)aminomethane, ≥99.5) and HCl 37% were purchased from Merck KGaA, Darmstadt, Germany. Chloroform, used as a solvent for the preparation of MAPLE targets, was also acquired from Merck.

### 3.2. Synthesis of Fe_3_O_4_@AN NPs

#### 3.2.1. Anthriscus sylvestris (AN) Extract

AN extract was obtained by using a microwave extractor (Ethos SEL-Milestone) equipped with a closed vessel (100 mL) and a temperature monitoring system. In our experiment, 5 g of whole plant material was mixed with 50 mL of selected solvent (ethanol:water = 70:30) and underwent microwave-assisted extraction for 1 h at 100 °C. After the extraction, the mixture was vacuum filtered through Whatman No. 1 filter paper and collected in a volumetric flask (45 mL of extract was recovered, i.e., 7.81 g extract/g plant material). The extract was further used in liquid form without performing a drying step in order to avoid sensitive compound degradation.

#### 3.2.2. Functionalized Fe_3_O_4_@AN NPs

Functionalized Fe_3_O_4_ nanoparticles are usually prepared by wet chemical precipitation from aqueous iron salt solutions by means of alkaline media, like HO^−^ and NH_3_ [71,72]. In this case, the AN-extract-functionalized Fe_3_O_4_ NPs were synthesized according to previously reported work [73,74] as follows: First, 500 mg of AN extract and 8 mL of NH_4_OH (25%) were added to 200 mL deionized water under stirring. Then, 1 g of FeCl_3_ (anhydrous) and 1.6 g of FeSO_4_·7H_2_O were dissolved in 200 mL of deionized water, and Fe^+3^/Fe^2+^ solution was dropped into the basic solution of AN extract. After co-precipitation, magnetite-*A. sylvestris* nanostructures (denoted as Fe_3_O_4_@AN) were washed several times with deionized water; after that, the NPs were washed three times with methanol and separated with a strong NdFeB permanent magnet.

### 3.3. MAPLE Experimental Conditions and Deposition of PLGA–Fe_3_O_4_@AN Thin Coatings

Detailed protocols for thin film assembly by MAPLE were addressed in [75] and are briefly described in the following. The MAPLE experimental procedure consists of laser irradiation of a cryogenic target by a pulsed laser beam. The MAPLE depositions of thin films were done by means of a KrF* (λ = 248 nm, τ_FWHM_ ≈ 25 ns) and an excimer laser source (COMPexPro 205, Coherent) in a stainless steel reaction chamber at room temperature. During all MAPLE experiments, the following parameters were kept constant: the target-to-substrate distance of 5 cm, a background pressure of 6 × 10^−3^ mbar, a laser fluence of 300 mJ/cm^2^, and a repetition rate of 15 Hz. The number of applied pulses for each deposition was 36,000, and the laser energy distribution into a laser spot was controlled with a laser beam homogenizer.

Before MAPLE deposition, the solution was prepared in a 3:1 mass ratio of PLGA:NPs suspended in 2 mL chloroform. The obtained solutions were immersed in liquid nitrogen (LN_2_) and operated as solid targets during laser deposition. During the laser deposition, the icy target was maintained at a temperature of ~173 K by continuous cooling with LN_2_. The target was rotated with 0.83 Hz to avoid laser-drilling.

Prior to the introduction in the deposition chamber, all substrates were successively cleaned with acetone, ethyl alcohol, and deionized water for 15 min in an ultrasonic bath. After this step, substrates were dried with a jet of high-purity nitrogen. The coatings were deposited onto double-side polished Si (100) substrates for FT-IR and SEM analyses and glass plates for antibacterial assays.

Drop-cast samples were prepared on Si (100) substrates to be compared with lased-obtained coatings in order to evaluate whether chemical changes appeared during the laser deposition.

### 3.4. Physicochemical Characterization

#### 3.4.1. High-Performance Liquid Chromatography (HPLC) with a Diode-Array Detector (DAD)

HPLC-DAD quantification of bioactive polyphenols from AN extracts was carried out using an HPLC system L-3000 (RIGOL Technologies, China) equipped with a Kinetex EVO C18 column (150 × 4.6 mm, particle size of 5 μm), and the injection volume was 20 µL. The solvents used were (A) 0.1 trifluoroacetic acid (TFA) in water and (B) 0.1 TFA in acetonitrile. The gradient elution was 2–100% B at 30 °C for 60 min, and the elution flow was set at 1 mL/min. Measurements were performed at λ max 250. The identification and quantification of compounds were done by comparison with standard spectra at each retention time. Stock solutions of reference substances were prepared at a concentration of 1000 μg/mL. For calibration curves, five concentrations were used (10, 50, 100, 200, and 400 μg/mL). The rest of the peaks were unidentified.

For the chemiluminescence (CL) assay, extracts were used undiluted. The assay was performed by using 200 µL 8 mM luminol, 50 µL 5 mM hydrogen peroxide, and 50 µL of the vegetal extract or standard in Tris-HCl buffer (0.2 M, pH 8.6). The buffer was obtained from Tris and HCl. The CL was measured on a Turner Biosystems Modulus.

The results were compared with the results obtained for two known antioxidants, rutin and ascorbic acid (99%, Sigma-Aldrich, MO, USA), at different concentrations. The following mathematical expression was used to obtain the antioxidant activity of each sample:

*AA* (%) = [(*I_0_ − I*)/*I_0_*] × 100 (1)

where *I_0_* represents the maximum CL intensity for the blank and *I* is the maximum CL intensity for a sample at *t* = 5 s after reaction initiation.

#### 3.4.2. Transmission Electron Microscopy

Relevant microstructural data on Fe_3_O_4_-based samples were obtained by transmission electron microscopy (TEM). TEM studies were achieved by using a Tecnai G2 F30 S-TWIN high-resolution transmission electron microscope from FEI Company (Hillsboro, OR, USA). The TEM apparatus was equipped with a selected area electron diffraction (SAED) instrument. The synthesized PLGA–Fe_3_O_4_@AN powders were dispersed in ethanol, sonicated for 15 min, placed onto carbon-coated copper grids, and dried at room temperature. The microscope was operated in transmission mode at 300 kV with a TEM point resolution of 2 Å and a line resolution of 1 Å.

#### 3.4.3. Infrared Microscopy (IR)

A Nicolet iN10 MX FT-IR microscope equipped with an MCT liquid-nitrogen-cooled detector was used to record IR mappings. All measurements were made in the range of 4000–700 cm^−1^. The spectral collection was conducted in reflection mode at 4 cm^−1^ resolution. For each spectrum, 32 individual scans were co-added and converted to absorbance by using the Omnic Picta dedicated software from Thermo Scientific (Waltham, MA, USA).

#### 3.4.4. Optical Microscopy

Images were recorded with a Zeiss optical microscope (Imager Z1m, Göttingen, Germany) equipped with an AxioCam MRc 5 (HR); the camera resolution was able to be varied from 1388 × 1040 to 4164 × 3120 pixels of 14 bits. For microscope analysis, the samples were placed in optical cells with 1 mm optical path.

### 3.5. Biological Evaluation

#### 3.5.1. In Vitro Cytotoxicity Assessment

Human colorectal adenocarcinoma cell line HT-29 was cultivated in direct contact with the PLGA–Fe_3_O_4_@AN composites or exposed to free-AN extract and bare Fe_3_O_4_ NP treatment. HT-29 cells were maintained in Dulbecco’s modified Eagle’s medium (DMEM, Sigma/Merck, Steinheim, Germany) supplemented with 10% FBS and 1% antibiotic–antimycotic solution (Sigma Sigma/Merck, Steinheim, Germany) under standard culture conditions (37 °C, 5% CO_2_). Before cell seeding, all the tested composites were sterilized by UV exposure. Afterward, HT-29 cells were seeded at the density of 10^4^ cells/cm^2^ on the surface of the PLGA–Fe_3_O_4_@AN composites or in 96-well cell culture plates. After 30 min, samples were immersed in complete culture media (DMEM supplemented with 10% FBS and 1% antibiotic–antimycotic solution) and further cultured at 37 °C in a humidified atmosphere containing 5% CO_2_. In the case of free-AN extracts or bare Fe_3_O_4_ NP treatment, cells were incubated for 24 h to allow adherence to the culture surface and afterward treated with free-AN extract and Fe_3_O_4_ NP solutions that were freshly prepared in culture medium. To determine the effect of PLGA–Fe_3_O_4_@AN composites and their components on HT-29 cell viability, the colorimetric 3-(4,5-dimethylthiazol-2-yl)-2,5-diphenyltetrazolium bromide (MTT) test was used. Briefly, after 24, 48, and 72 h, the culture media were discarded and replaced with a 1 mg MTT/mL solution, freshly prepared in serum-free culture media. After 4 h of incubation with the MTT solution in the dark and standard culture conditions, the resulting formazan crystals were dissolved in isopropanol. The absorbance of the resulting solution was measured with the multimodal FlexStation III microplate reader (Molecular Devices, San Jose, CA, USA) at 550 nm. HT-29 cell membrane integrity in contact with PLGA–Fe_3_O_4_@AN composites or exposed to free-AN extract and Fe_3_O_4_ NPs was evaluated by quantifying the levels of lactate dehydrogenase (LDH) released in the culture media using the TOX7 kit. For this, media samples were collected after 24, 48, and 72 h and mixed with the kit components according to the manufacturer’s instruction. The absorbance of the resulting solution was measured at 490 nm using the multimodal FlexStation III microplate reader (Molecular Devices, San Jose, CA, USA). All the presented experiments were performed in triplicate (*n* = 3). The obtained results were then statistically analyzed using Graph Pad 6 software (one-way ANOVA, Bonferroni test). All the data are expressed as mean ± standard error of the mean. A *p*-value of ≤0.05 was considered statistically significant.

#### 3.5.2. Antimicrobial Activity

The antimicrobial potential of PLGA–Fe_3_O_4_@AN coatings was assessed against opportunistic microbial pathogens (laboratory strains): *Staphylococcus aureus* ATCC 23235 (a Gram-positive model strain that is clinically relevant and biofilm-forming), *Escherichia coli* ATCC 2592 (a Gram-negative model strain that is clinically relevant and biofilm-forming) and *Candida albicans* ATCC 10231 (a fungal model strain that is clinically relevant and biofilm-forming). The assay followed a protocol adopted from [76,77]. Briefly, microbial strains grown on nutritive agar for 20 h at 37 °C were used to obtain microbial suspensions of 0.5 McFarland density (1.5 × 10^8^ CFU (colony-forming units)/mL), prepared in sterile buffered saline solution. Sterile samples (squares of 1 cm with 1 cm of glass containing the MAPLE-deposited coatings on both sides) of the obtained nanostructured coatings were aseptically transferred in sterile six-well plates, and 2 mL of Luria broth for bacteria or YPG (yeast peptone glucose) for *C. albicans* were added in each well containing one sterile specimen of one type of coating. Then, 20-µL aliquots of the prepared microbial suspensions were added in the respective wells, and plates were incubated at 37 °C for various time intervals (24, 48, and 72 h) in order to evaluate the ability of microorganisms to colonize and develop biofilms on the tested coatings. For the biofilm evaluation, after each 24 h of incubation, the specimens containing attached bacteria were gently washed with sterile phosphate-buffered saline (PBS) to remove unattached cells and transferred in sterile culture media to ensure the growth of biofilm-embedded cells only and provide them with fresh nutrients. After each incubation interval, the specimens were washed with 1 mL of sterile PBS in order to remove unattached bacteria and placed in a sterile tube containing 1 mL of sterile saline water. Tubes were then vigorously vortexed for 20 s and sonicated for 10 s to determine the detachment of biofilm-embedded bacteria. The resulting suspensions containing biofilm-detached bacteria were utilized to perform serial dilutions that were inoculated in triplicate on nutritive agar plates. The inoculated plates were incubated 24 h at 37 °C to allow the growth of inoculated microbial cells, and then the number of CFU (colony-forming units) was calculated for each sample. The CFU/mL values were represented in a logarithmic scale, and they reflect the number of microbial cells which attached to the tested coatings and developed biofilms at various time intervals.

## 4. Conclusions

In this study, we confirmed that MAPLE represents a promising deposition technique for obtaining nanocomposite coatings with multifunctional activity, namely antimicrobial and cytotoxic activity, against tumor cells.

Previously reported antimicrobial and antitumor efficiency of the AN extract is maintained within the developed coatings, with the developed nanostructured thin films ensuring their controlled release for at least 3 days.

Such thin coatings represent innovative and useful solutions for the development of natural and bioactive materials with multifunctional applications.

## Figures and Tables

**Table 1:** HPLC-DAD identification and quantification of some polyphenols from *Anthriscus sylvestris* extracts.

Peak	Compound	T_R_ *		Concentration		
	(mg/L)	(min)		(mg/L)		
			Extract 1	Extract 2	Extract 3	Average
1	Tannic acid	2.496	3.35	3.44	3.47	3.42 ± 0.06
2	Caffeic acid	19.573	13.48	12.95	14.19	13.54 ± 0.63
3	Chlorogenic acid	20.88	373.16	370.79	377.36	373.77 ± 3.33
4	Epicatechin (-)	22.913	402.46	410.05	419.68	410.73 ± 8.63
5	Delphinidin	23.262	33.39	30.09	31.62	31.7 ± 1.65
6	Daidzein	26.719	10.90	10.74	11.26	10.97 ± 0.27
7	Rutin	30.302	172.01	171.37	173.10	172.16 ± 0.87
8	Malvidin	33.098	496.85	485.31	497.83	493.33 ± 6.96
9	Naringenin	39.087	16.18	13.64	17.87	15.9 ± 2.13

* Retention time (T_R_) error of mean for compounds was ± 0.0001–0.2 min.

## References

[1] World Health Organization. https://www.who.int/news-room/fact-sheets/detail/cancer.

[2] Fernandes R.T.S., França E.L., Triches D.L.G.F., Fujimori M., Machi P.G.F., Massmman P.F., Tozetti I.A., Honorio-França A.C. (2019). Nanodoses of melatonin induces apoptosis on human breast cancer cells co-cultured with colostrum cells. Biointerface Res. Appl. Chem..

[3] Howlader N.N.A., Krapcho M., Garshell J., Miller D., Altekruse S.F., Kosary C.L., Yu M., Ruhl J., Tatalovich Z., Mariotto A. (2015). SEER Cancer Statistics Review, 1975-2012.

[4] Ferlay J., Soerjomataram I., Dikshit R., Eser S., Mathers C., Rebelo M., Parkin D.M., Forman D., Bray F. (2015). Cancer incidence and mortality worldwide: Sources, methods and major patterns in GLOBOCAN 2012. Int. J. Cancer.

[5] Faden A.A. (2016). The potential role of microbes in oncogenesis with particular emphasis on oral cancer. Saudi Med. J..

[6] Song S., Vuai M.S., Zhong M. (2018). The role of bacteria in cancer therapy-enemies in the past, but allies at present. Infect. Agents Cancer.

[7] Golemis E.A., Scheet P., Beck T.N., Scolnick E.M., Hunter D.J., Hawk E., Hopkins N. (2018). Molecular mechanisms of the preventable causes of cancer in the United States. Genes Dev..

[8] Antonic V., Stojadinovic A., Kester K.E., Weina P.J., Brücher B.L.D.M., Protic M., Avital I., Izadjoo M. (2013). Significance of Infectious Agents in Colorectal Cancer Development. J. Cancer.

[9] Martin H.M., Campbell B.J., Hart C.A., Mpofu C., Nayar M., Singh R., Englyst H., Williams H.F., Rhodes J.M. (2004). Enhanced *Escherichia coli* adherence and invasion in Crohn’s disease and colon cancer. Gastroenterology.

[10] Maddocks O.D., Short A.J., Donnenberg M.S., Bader S., Harrison D.J. (2009). Attaching and effacing *Escherichia coli* downregulate DNA mismatch repair protein in vitro and are associated with colorectal adenocarcinomas in humans. Plos One.

[11] Buc E., Dubois D., Sauvanet P., Raisch J., Delmas J., Darfeuille-Michaud A., Pezet D., Bonnet R. (2013). High prevalence of mucosa-associated *E. coli* producing cyclomodulin and genotoxin in colon cancer. PLoS ONE.

[12] Arthur J.C., Perez-Chanona E., Mühlbauer M., Tomkovich S., Uronis J.M., Fan T.J., Campbell B.J., Abujamel T., Dogan B., Rogers A.B. (2012). Intestinal inflammation targets cancer-inducing activity of the microbiota. Science.

[13] Markou P., Apidianakis Y. (2014). Pathogenesis of intestinal Pseudomonas aeruginosa infection in patients with cancer. Front. Cell. Infect. Microbiol..

[14] Ramirez-Garcia A., Rementeria A., Aguirre-Urizar J.M., Moragues M.D., Antoran A., Pellon A., Abad-Diaz-de-Cerio A., Hernando F.L. (2016). Candida albicans and cancer: Can this yeast induce cancer development or progression?. Crit. Rev. Microbiol..

[15] Raphel J., Holodniy M., Goodman S.B., Heilshorn S.C. (2016). Multifunctional coatings to simultaneously promote osseointegration and prevent infection of orthopaedic implants. Biomaterials.

[16] Martín Ortega A.M., Segura Campos M.R., Campos M.R.S. (2019). Chapter 5-Medicinal Plants and Their Bioactive Metabolites in Cancer Prevention and Treatment. Bioactive Compounds.

[17] Raskin I., Ribnicky D.M., Komarnytsky S., Ilic N., Poulev A., Borisjuk N., Brinker A., Moreno D.A., Ripoll C., Yakoby N. (2002). Plants and human health in the twenty-first century. Trends Biotechnol..

[18] Radwan A., Khalid M., Amer H., Alotaibi M. (2019). Anticancer and molecular docking studies of some new pyrazole-1-carbothioamide nucleosides. Biointerface Res. Appl. Chem..

[19] Fabricant D.S., Farnsworth N.R. (2001). The value of plants used in traditional medicine for drug discovery. Environ. Health Perspect..

[20] Alviano D.S., Alviano C.S. (2009). Plant extracts: Search for new alternatives to treat microbial diseases. Curr. Pharm. Biotechnol..

[21] Harikrishnan R., Balasundaram C., Goyal M.R., Suleria H.A.R., Harikrishnan R. (2020). Potential of Herbal Extracts and Bioactive Compounds for Human Healthcare. The Role of Phytoconstitutents in Health Care: Biocompounds in Medicinal Plant.

[22] Nwonu C., Ilesanmi O., Agbedahunsi J., Nwonu P.J.I.J.o.H.M. (2019). Natural products as veritable source of novel drugs and medicines: A review. Int. J. Herb. Med..

[23] Ghosh A., Das B.K., Roy A., Mandal B., Chandra G. (2008). Antibacterial activity of some medicinal plant extracts. J. Nat. Med..

[24] Hassan N., Wali H., Faiz-Ul-Hassan, Shuaib M., Nisar M., Din M.U., Wadood S.F., Shah S.S., Ali M., Shah M. (2018). Ethnobotanical study of medicinal plants used for primary health care in Shergarh, District Mardan, Pakistan. Biointerface Res. Appl. Chem..

[25] Dias-Souza M.V., Dias C.G., Ferreira-Marcal P.H. (2018). Interactions of natural products and antimicrobial drugs: Investigations of a dark matter in chemistry. Biointerface Res. Appl. Chem..

[26] Vaishali S., Deepika R., Anuj K., Himanshu C. (2019). Formulation and Evaluation of Herbal Tablet Containing *Terminalia Chebula* Extract. Lett. Appl. Nanobioscience.

[27] Sakarkar D., Deshmukh V. (2011). Ethnopharmacological review of traditional medicinal plants for anticancer activity. Int. J. Pharm. Tech. Res..

[28] Filippi A., Maru N., Chifiriuc M.C., Grigore R., Ganea C., Mocanu M.M. (2019). Anticancer effects of curcumin in luminal B and HER2 breast cancer cell line models. Rom. Biotechnol. Lett..

[29] Rehman S.S., Ashraf A., Nazli Z.I.H., Kausar A., Rafique N., Perveen S., Majeed H.N., Shafiq N. (2019). Anticancer Activity of Natural Bioactive Compounds against Human Carcinoma Cell Lines—A mini review. Rom. Biotechnol. Lett..

[30] Mathiyalagan S., Mandal B.K. (2019). Preparation of metal doped quercetin nanoparticles, characterization and their stability study. Lett. Appl. Nanobioscience..

[31] Johnson M. (2018). Antifungal Activity of Different Essential Oils. https://digitalcommons.murraystate.edu/postersatthecapitol/2018/PLTW/5/.

[32] Bahramian G., Golestan L., Khosravi-Darani K. (2018). Antimicrobial and antioxidant effect of nanoliposomes containing zataria multiflora boiss essential oil on the rainbow trout fillets during refrigeration. Biointerface Res. Appl. Chem..

[33] Marutescu L., Popa M., Surugiu M., Pircalabioru G.G., Craciun N. (2019). Physiological profile of microbial communities associated with some plant aquatic species. Rom. Biotechnol. Lett..

[34] Otuechere C.A., Durugbo E.U., Adesanya O., Omotolani F.O., Osho A. (2019). Essential Oil of Alchornea Laxiflora (benth): Phytochemical, Antimicrobial and Toxicity Evaluations. Lett. Appl. Nanobioscience.

[35] Khammee T., Phoonan W., Ninsuwan U., Jaratrungtawee A., Kuno M. (2019). Volatile constituents, in vitro and in silico anti-hyaluronidase activity of the essential oil from *Gardenia carinata* wall. ex roxb. flowers. Biointerface Res. Appl. Chem..

[36] Mittal R.P., Rana A., Jaitak V. (2019). Essential Oils: An Impending Substitute of Synthetic Antimicrobial Agents to Overcome Antimicrobial Resistance. Curr. Drug Targets.

[37] Estrela J.M., Mena S., Obrador E., Benlloch M., Castellano G., Salvador R., Dellinger R.W. (2017). Polyphenolic Phytochemicals in Cancer Prevention and Therapy: Bioavailability versus Bioefficacy. J. Med. Chem..

[38] Collins D., Hogan A.M., Winter D.C. (2011). Microbial and viral pathogens in colorectal cancer. Lancet Oncol..

[39] Lewandowska U., Szewczyk K., Hrabec E., Janecka A., Gorlach S. (2013). Overview of Metabolism and Bioavailability Enhancement of Polyphenols. J. Agric. Food Chem..

[40] Plunkett G.M., Soltis D.E., Soltis P.S. (1996). Evolutionary patterns in Apiaceae: Inferences based on matK sequence data. Syst. Bot..

[41] Kozawa M., Baba K., Matsuyama Y., Kido T., Sakai M., Takemoto T. (1982). Components of the root of Anthriscus sylvestris HOFFM. II. Insecticidal activity. Chem. Pharm. Bull..

[42] Cho E.J., Choi J.M., Kim H.M., Choi K., Ku J., Park K.-W., Kim J., Lee S. (2013). Antibacterial activity and protective effect against gastric cancer by Anthriscus sylvestris fractions. Hortic. Environ. Biotechnol..

[43] Ikeda R., Nagao T., Okabe H., Nakano Y., Matsunaga H., Katano M., Mori M. (1998). Antiproliferative constituents in Umbelliferae plants. III. Constituents in the root and the ground part of Anthriscus sylvestris Hoffm. Chem. Pharm. Bull..

[44] Pérez-Artacho B., Gallardo V., Ruiz M.A., Arias J.L. (2012). Maghemite/poly (D, L-lactide-co-glycolyde) composite nanoplatform for therapeutic applications. J. Nanoparticle Res..

[45] Prodan A.M., Beuran M., Turculet C.S., Popa M., Andronescu E., Bleotu C., Raita S.M., Soare M., Lupescu O. (2018). In vitro evaluation of glycerol coated iron oxide nanoparticles in solution. Rom. Biotechnol. Lett..

[46] Sala F., Boldea M., Botau D., Pirvulescu A., Gergen I. (2019). Fe_3_O_4_-water based magnetic nanofluid influence on weight loss of wheat seedlings under controlled conditions. Rom. Biotechnol. Lett..

[47] Taresco V., Francolini I., Padella F., Bellusci M., Boni A., Innocenti C., Martinelli A., D’Ilario L., Piozzi A. (2015). Design and characterization of antimicrobial usnic acid loaded-core/shell magnetic nanoparticles. Mater. Sci. Eng. C Mater. Biol. Appl..

[48] Hashemi E., Mahdavi H., Khezri J., Razi F., Shamsara M., Farmany A. (2019). Enhanced Gene Delivery in Bacterial and Mammalian Cells Using PEGylated Calcium Doped Magnetic Nanograin. Int. J. Nanomed..

[49] Negut I., Grumezescu V., Dorcioman G. (2017). Progress of nanoparticles research in cancer therapy and diagnosis. Nanostructures for Cancer Therapy.

[50] Sharma S., Parmar A., Kori S., Sandhir R. (2016). PLGA-based nanoparticles: A new paradigm in biomedical applications. Trac Trends Anal. Chem..

[51] Arzani H., Adabi M., Mosafer J., Dorkoosh F., Khosravani M., Maleki H., Nekounam H., Kamali M. (2019). Preparation of curcumin-loaded PLGA nanoparticles and investigation of its cytotoxicity effects on human glioblastoma U87MG cells. Biointerface Res. Appl. Chem..

[52] Albinali K.E., Zagho M.M., Deng Y., Elzatahry A.A. (2019). A perspective on magnetic core–shell carriers for responsive and targeted drug delivery systems. Int. J. Nanomed..

[53] Malekpour M.R., Naghibzadeh M., Najafabadi M.R.H., Esnaashari S.S., Adabi M., Mujokoro B., Khosravani M., Adabi M. (2018). Effect of various parameters on encapsulation efficiency of mPEG-PLGA nanoparticles: Artificial neural network. Biointerface Res. Appl. Chem..

[54] Grumezescu V., Negut I. (2019). Nanocoatings and thin films. Materials for Biomedical Engineering: Inorganic Micro- and Nanostructures.

[55] Grumezescu V., Negut I., Gherasim O., Birca A.C., Grumezescu A.M., Hudita A., Galateanu B., Costache M., Andronescu E., Holban A.M. (2019). Antimicrobial applications of MAPLE processed coatings based on PLGA and lincomycin functionalized magnetite nanoparticles. Appl. Surf. Sci..

[56] Domitrovic R. (2011). The molecular basis for the pharmacological activity of anthocyans. Curr. Med. Chem..

[57] Stoner G.D., Wang L.-S., Sardo C., Zikri N., Hecht S.S., Mallery S.R. (2010). Cancer prevention with berries: Role of anthocyanins. Bioactive Compounds and Cancer.

[58] Choi E.J., Kim G.-H. (2013). Antiproliferative activity of daidzein and genistein may be related to ERα/c-erbB-2 expression in human breast cancer cells. Mol. Med. Rep..

[59] Raeisi S., Chavoshi H., Mohammadi M., Ghorbani M., Sabzichi M., Ramezani F. (2019). Naringenin-loaded nano-structured lipid carrier fortifies oxaliplatin-dependent apoptosis in HT-29 cell line. Process Biochem..

[60] Shay J., Elbaz H.A., Lee I., Zielske S.P., Malek M.H., Hüttemann M. (2015). Molecular mechanisms and therapeutic effects of (−)-epicatechin and other polyphenols in cancer, inflammation, diabetes, and neurodegeneration. Oxidative Med. Cell. Longev..

[61] Huang S., Wang L.-L., Xue N.-N., Li C., Guo H.-H., Ren T.-K., Zhan Y., Li W.-B., Zhang J., Chen X.-G. (2019). Chlorogenic acid effectively treats cancers through induction of cancer cell differentiation. Theranostics.

[62] Guan D., Gao Z., Yang W., Wang J., Yuan Y., Wang B., Zhang M., Liu L. (2013). Hydrothermal synthesis of carbon nanotube/cubic Fe_3_O_4_ nanocomposite for enhanced performance supercapacitor electrode material. Mater. Sci. Eng. B.

[63] Ficai D., Grumezescu V., Fufă O.M., Popescu R.C., Holban A.M., Ficai A., Grumezescu A.M., Mogoanta L., Mogosanu G.D., Andronescu E. (2018). Antibiofilm coatings based on PLGA and nanostructured cefepime-functionalized magnetite. Nanomaterials.

[64] Grumezescu A.M., Andronescu E., Holban A.M., Ficai A., Ficai D., Voicu G., Grumezescu V., Balaure P.C., Chifiriuc C.M. (2013). Water dispersible cross-linked magnetic chitosan beads for increasing the antimicrobial efficiency of aminoglycoside antibiotics. Int. J. Pharm..

[65] Grumezescu V., Socol G., Grumezescu A.M., Holban A.M., Ficai A., Truşcǎ R., Bleotu C., Balaure P.C., Cristescu R., Chifiriuc M.C. (2014). Functionalized antibiofilm thin coatings based on PLA–PVA microspheres loaded with usnic acid natural compounds fabricated by MAPLE. Appl. Surf. Sci..

[66] Grumezescu V., Holban A.M., Iordache F., Socol G., Mogoşanu G.D., Grumezescu A.M., Ficai A., Vasile B.Ş., Truşcă R., Chifiriuc M.C. (2014). MAPLE fabricated magnetite@ eugenol and (3-hidroxybutyric acid-co-3-hidroxyvaleric acid)–polyvinyl alcohol microspheres coated surfaces with anti-microbial properties. Appl. Surf. Sci..

[67] Zhang H.-M. (2012). Identification of ginseng and its counterfeit by laser Raman spectroscopy. Spectrosc. Spectr. Anal..

[68] Liakos I., Grumezescu A.M., Holban A.M. (2014). Magnetite nanostructures as novel strategies for anti-infectious therapy. Molecules.

[69] Grumezescu V., Gherasim O., Negut I., Banita S., Holban A.M., Florian P., Icriverzi M., Socol G. (2019). Nanomagnetite-embedded PLGA Spheres for Multipurpose Medical Applications. Materials.

[70] Marková Z., Šišková K., Filip J., Šafářová K., Prucek R., Panáček A., Kolář M., Zbořil R. (2012). Chitosan-based synthesis of magnetically-driven nanocomposites with biogenic magnetite core, controlled silver size, and high antimicrobial activity. Green Chem..

[71] Grumezescu A.M., Holban A., Andronescu E., Ficai A., Bleotu C., Chifiriuc M.C. (2012). Water dispersible metal oxide nanobiocomposite as a potentiator of the antimicrobial activity of kanamycin. Lett. Appli. NanoBioScience.

[72] Grumezescu A.M., Andronescu E., Ficai A., Ficai D., Huang K.S., Gheorghe I., Chifiriuc C.M. (2012). Water soluble magnetic biocomposite with potential applications for the antimicrobial therapy. Biointerface Res. Appl. Chem..

[73] Grumezescu A.M., Cristescu R., Chifiriuc M., Dorcioman G., Socol G., Mihailescu I., Mihaiescu D.E., Ficai A., Vasile O., Enculescu M. (2015). Chrisey, D.B. Fabrication of magnetite-based core–shell coated nanoparticles with antibacterial properties. Biofabrication.

[74] Chifiriuc M.C., Grumezescu A.M., Andronescu E., Ficai A., Cotar A.I., Grumezescu V., Bezirtzoglou E., Lazar V., Radulescu R. (2013). Water dispersible magnetite nanoparticles influence the efficacy of antibiotics against planktonic and biofilm embedded Enterococcus faecalis cells. Anaerobe.

[75] Stan M.S., Constanda S., Grumezescu V., Andronescu E., Ene A.M., Holban A.M., Vasile B.S., Mogoantă L., Bălşeanu T.-A., Mogoşanu G.D. (2016). Thin coatings based on ZnO@ C18-usnic acid nanoparticles prepared by MAPLE inhibit the development of Salmonella enterica early biofilm growth. Appl. Surf. Sci..

[76] Grumezescu V., Negut I., Grumezescu A.M., Ficai A., Dorcioman G., Socol G., Iordache F., Truşcă R., Vasile B.S., Holban A.M. (2018). MAPLE fabricated coatings based on magnetite nanoparticles embedded into biopolymeric spheres resistant to microbial colonization. Appl. Surf. Sci..

[77] Negut I., Grumezescu V., Ficai A., Grumezescu A.M., Holban A.M., Popescu R.C., Savu D., Vasile B.S., Socol G. (2018). MAPLE deposition of Nigella sativa functionalized Fe3O4 nanoparticles for antimicrobial coatings. Appl. Surf. Sci..

